# Friends against the Foe: Synergistic Photothermal and Photodynamic Therapy against Bacterial Infections

**DOI:** 10.3390/pharmaceutics15041116

**Published:** 2023-03-31

**Authors:** Atanu Naskar, Kwang-sun Kim

**Affiliations:** Department of Chemistry and Chemistry Institute for Functional Materials, Pusan National University, Busan 46241, Republic of Korea; atanunaskar@pusan.ac.kr

**Keywords:** photodynamic therapy, photothermal therapy, synergistic effect, antibacterial, multidrug-resistant bacteria

## Abstract

Multidrug-resistant (MDR) bacteria are rapidly emerging, coupled with the failure of current antibiotic therapy; thus, new alternatives for effectively treating infections caused by MDR bacteria are required. Hyperthermia-mediated photothermal therapy (PTT) and reactive oxygen species (ROS)-mediated photodynamic therapy (PDT) have attracted extensive attention as antibacterial therapies owing to advantages such as low invasiveness, low toxicity, and low likelihood of causing bacterial resistance. However, both strategies have notable drawbacks, including the high temperature requirements of PTT and the weak ability of PDT-derived ROS to penetrate target cells. To overcome these limitations, a combination of PTT and PDT has been used against MDR bacteria. In this review, we discuss the unique benefits and limitations of PTT and PDT against MDR bacteria. The mechanisms underlying the synergistic effects of the PTT–PDT combination are also discussed. Furthermore, we introduced advancements in antibacterial methods using nano-based PTT and PDT agents to treat infections caused by MDR bacteria. Finally, we highlight the existing challenges and future perspectives of synergistic PTT–PDT combination therapy against infections caused by MDR bacteria. We believe that this review will encourage synergistic PTT- and PDT-based antibacterial research and can be referenced for future clinical applications.

## 1. Introduction

The prevalence of multidrug-resistant (MDR) bacteria and their infections in humans has frightened the global health system. Identifying them is a critical issue that must be addressed to ensure that people live in safe and healthy environments [1]. The overall number of deaths from bacterial infections is expected to increase to 10 million per year by 2050, illustrating the severity of MDR bacterial infections [2]. Currently, it is feasible that a bacterial infection might not respond to current antibiotic therapies. The overuse or misuse of conventional antibiotics is to blame for this unbelievable situation [3]. Notably, bacteria develop resistance to antibiotics at a considerably higher rate than that at which new antibiotics are discovered.

Bacteria have evolved various protection mechanisms against antibiotics over time [4,5]. These include the development of the drug efflux pump in bacteria for allowing drugs to be expelled outside of cells, evolved abilities to break down antibiotics using enzymes produced by bacteria, modifications of bacterial metabolic pathways to protect them from antibiotics, formations of biofilms, and structured colonization of bacterial cells with a self-produced extracellular polymeric substance matrix. Antibiotics cannot function through this protective biofilm matrix and are captured in the matrix [6]. Furthermore, bacteria enclosed in the biofilm matrix can efficiently communicate and transfer antibiotic-resistance genes among all bacteria, making it more difficult to kill using antibiotics from outside the biofilms. Unsurprisingly, approximately 80% of chronic and recurrent infections in the human body are associated with the formation of dense bacterial biofilms [6]. Therefore, an urgent need exists for thorough and methodical research on techniques for preventing MDR bacteria from rapidly multiplying after infecting humans, in addition to techniques for eliminating bacteria.

Research has explored various alternatives to conventional antibiotics to manage this constantly hazardous and demanding scenario with varying degrees of effectiveness. However, these alternatives are not perfect solutions for overcoming the limitations of antibiotic therapies. For instance, naturally occurring antimicrobial compounds have been investigated; however, they are expensive to use [7]. Antimicrobial peptides (AMPs) have been characterized and suggested as promising alternatives owing to their potent antibacterial activity and species selectivity; however, they are unstable and very vulnerable to proteolysis, limiting their potential for use [8]. To overcome these limitations, molecules designed to mimic the properties of antimicrobial peptides have resulted in synthetic mimics of antimicrobial peptides [9]. These are polymeric mimics of AMPs, peptidomimetic oligomers, or small molecules. Most are prepared with the aim of overcoming the issues of protease lability, toxicity, and high costs in manufacturing AMPs. However, optimizing these materials to achieve controlled bioactivity remains challenging. Quaternary ammonium compounds have been used because of their antibacterial activity; however, their bacterial resistance after long-term use needs to be resolved [10]. Metal-based nanoparticles have recently been extensively developed in view of their well-received antibacterial activity, but their toxicity to mammalian cells is yet to be fully resolved [11].

Following the above trends, phototherapy has emerged as a promising strategy against bacteria owing to its excellent antibacterial activity, non-invasive nature, and fewer chances of bacteria generating bacterial resistance [12]. Hyperthermia-mediated photothermal therapy (PTT) and reactive oxygen species (ROS)-mediated photodynamic therapy (PDT) are the two most widely utilized phototherapies against bacterial infections. The basic mechanism of PTT depends on the light-to-heat conversion ability of photothermal agents (PTAs) after irradiation with desirable light sources. PTAs damage and kill bacteria through various processes (for example, membrane disruption and intracellular component disintegration of bacteria) [13]. Owing to their non-invasive nature, the chances of bacteria becoming resistant to this process are very unlikely relative to those with antibiotic therapies. Moreover, the usual light source utilized in this strategy is near-infrared (NIR) light between 700 and 1400 nm, which shows good tissue penetration ability owing to its longer wavelength. Accordingly, it can influence deep tissues for photothermal antibacterial treatment [14]. Additionally, a biofilm structure can be damaged by hyperthermia, facilitating the penetration of antibacterial agents through the biofilm matrix and efficiently killing bacteria [15]. This localized hyperthermia-mediated strategy for broad-spectrum antibacterial activity has potential clinical applications. However, it also has certain limitations, such as requiring high temperatures (≥60 °C), long-term laser exposures, and a high PTA dosage, which may cause inevitable thermal damage to normal tissues [16].

PDT is another non-invasive therapy. It utilizes a light source to activate photoresponsive materials to generate ROS. The ROS inflict oxidative damage on intracellular components of bacteria and eventually kill them [17]. Similar to PTT, the chance of bacteria becoming resistant to PDT is very unlikely, owing to its light source mechanism. Over the years, the biocompatibilities of the photoresponsive materials for PDT have been optimized with various types of functionalization for more productive and effective antibacterial activity, thereby providing greater potential for clinical applications [12]. However, limitations such as the weak penetration ability of the short-wavelength light for PDT [18] and the lifetime of ROS [19] need to be addressed before the strategy can be fully utilized in clinics.

In this regard, a combination of PTT and PDT seems promising, as both strategies can complement each other’s limitations [20]. The PTT and PDT synergistic therapy combination not only maintains the beneficial properties of each strategy but also compensates for the deficiencies of each strategy. The high-temperature requirement of PTT and the weak penetration ability of PDT can be easily overcome if PTT and PDT are synergistically utilized. Therefore, in this review, we briefly describe the advantages and limitations of using PTT and PDT individually, along with their antibacterial mechanisms. Following this, the importance of synergistic PTT and PDT and why they complement each other is discussed. Various advances in antibacterial therapy based on synergistic PTT and PDT are extensively discussed. Finally, future perspectives on the synergistic potential are discussed.

## 2. Photothermal Therapy (PTT) and Photodynamic Therapy (PDT)

### 2.1. Photothermal Therapy (PTT)

PTT is a therapeutic method in which light energy is converted into heat energy after PTAs are irradiated by external light sources, such as NIR [20]. This converted heat energy can effectively kill bacteria through a variety of thermal effects, such as cell membrane rupture, cell fluid evaporation, protein/enzyme degeneration, and cell hollowing. Previously, anti-cancer applications were the most used fields for PTT [21]. However, researchers have also utilized PTT in various ways for antibacterial activity and wound healing [13,20]. PTT is a non-invasive technique with low side effects and high specificity. Owing to these beneficial attributes, PTT has emerged as a promising strategy for combatting MDR pathogenic infections.

#### 2.1.1. Mechanism of PTT

**Figure 1:**
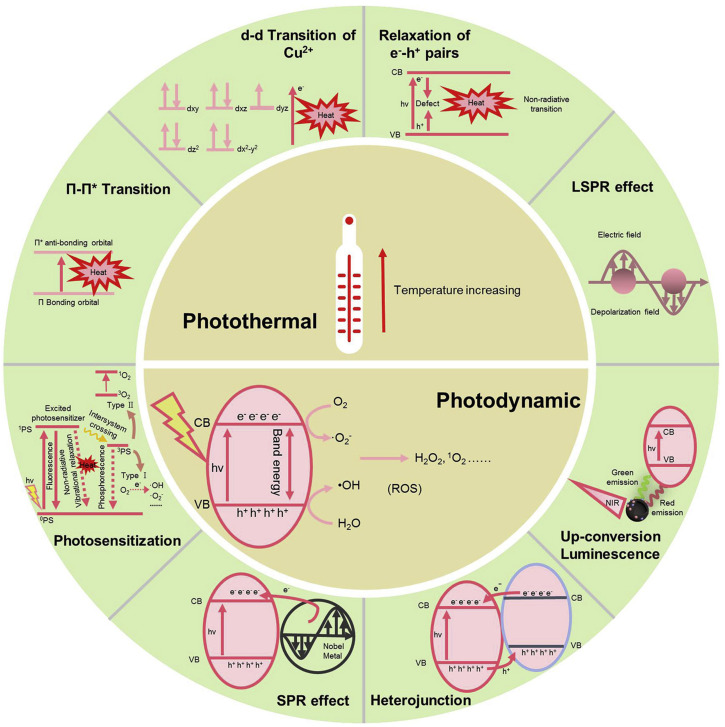
Mechanisms of photothermal therapy (PTT) and photodynamic therapy (PDT). Reproduced with permission from Ref. [12]. Copyright 2020 Elsevier. LSPR: localized surface plasmon resonance, SPR: surface plasmon resonance, CB: conduction band, VB: valence band.

The working mechanism of PTT involves the conversion of light energy into heat energy for use in the surrounding environment by photothermal materials. Owing to the distinct photophysical characteristics of various materials, it is evident that photothermal conversion mechanisms are different for different materials. In this section, the photothermal conversion mechanisms (Figure 1) of all the photothermal materials are broadly described.

PTTs using noble metal (for example, Ag and Au)-based nanomaterials are well-known [22], and their excellent photothermal properties can be attributed to the localized surface plasmon resonance (LSPR) effect of the nanomaterials [23]. Free electrons from the noble metallic nanoparticle surface are excited after the nanoparticles absorb the energy of photons at the appropriate wavelengths. The conduction band electrons then start to vibrate collectively at the same frequency. The LSPR effect is the term applied to this phenomenon [24]. LSPR can decay both radiatively and non-radiatively. The plasmonic enhancement of the electric field in the near-field regime is mainly governed by the radiative decay process, whereas the formation of hot electrons is directed by the non-radiative decay process via intra- or inter-band transitions. As light absorption (for example, NIR light) can be increased by modifying a particle size or structure, the LSPR effect is significantly correlated with several characteristics, including particle morphology, size, and composition [25]. The plasma coupling effect is another approach for enhancing the LSPR effect. Although noble metal (Ag and Au)-based nanomaterials are mostly used for the plasmonic effect, some materials, such as Al, Cu, Co, Ni, and CuS, have also been investigated for the same purpose [23].

Another photothermal conversion mechanism comprises the generation and relaxation of electron (e^−^)–hole (h^+^) pairs (Figure 1); these often occur in semiconductors [26]. In this process, the semiconductor absorbs photons to produce active e^−^–h^+^ pairs after being irradiated with light energy (the energy should be equal to or greater than the band gap energy of the semiconductor). After light irradiation, electrons are generated in the conduction band (CB), followed by electronic vacancies or holes in the valence band (VB). At this point, either the radiative (photons) or non-radiative (phonons) process is used for the subsequent relaxation from the higher excited states to the lower-energy states. This non-radiative process releases heat, resulting in a thermal (vibrational) energy increment of the lattice, which can be measured as an increase in their temperature.

Lattice vibrations are another photothermal conversion mechanism by which carbon and polymer-based materials exhibit excellent photothermal properties [26]. In this mechanism, less tightly held electrons in ᴨ bonds from the ᴨ orbital can be easily excited to the ᴨ* orbital with lower energy input. Notably, the light-irradiated excitation of electrons (ᴨ→ᴨ*) induces a strong absorption in the NIR region. The excited electrons released the absorbed energy as heat during their return to the ground state, resulting in an increase in the temperature (Figure 1).

#### 2.1.2. Advantages of PTT

PTT has emerged as a potential solution for treating MDR pathogens and a viable alternative to antibiotics [13,20,27] owing to the following advantages: (1) broad-spectrum antibacterial effects for both Gram-negative (G−) bacteria and Gram-positive (G+) bacteria irrespective of the membrane structure, owing to the penetration ability of the light sources (for example NIR) utilized in PTT; (2) a good tissue penetration ability without causing tissue damage based on the commonly utilized NIR light source (700–1400 nm), which enhances the chances for successful bacterial treatments (even in deep tissues); (3) localized hyperthermia for the antibacterial activity to reduce the risk of damaging normal cells; (4) facilitation of antibacterial agent penetration inside the biofilm via hyperthermia; and (5) a non-invasive and non-contact mechanism which minimizes the opportunity for bacteria to obtain resistance against the therapy. From the above features, it is easy to understand why PTT-mediated therapy for combatting MDR pathogens is gathering significant attention.

#### 2.1.3. Limitations of PTT

Despite the considerable interest in PTT, significant obstacles remain before PTT can be fully employed for practical applications. First, PTT usually requires high temperatures (≥60 °C) to kill bacteria [20]. Such prolonged hyperthermia kills bacterial cells and thermally harms the normal tissues around bacterial infection sites [16]. Therefore, a more strategic design is necessary to optimize the conditions, for example, with shorter treatment times at lower temperatures (~50 °C). Additionally, normal tissues may be critically affected by the necessity for a high excitation light power and high PTA dosage; these aspects require quick attention to ensure safer and more efficient PTT.

### 2.2. Photodynamic Therapy (PDT)

PDT combines photoresponsive materials such as photocatalysts and photosensitizers (PSs) and a light source to kill bacterial cells [17]. Currently, PDT has mainly been applied to cancers as a clinical therapeutic approach to treating non-invasive tumors. It is regarded as a major step in anti-cancer applications, including surgery, chemotherapy, and radiotherapy [28]. Additionally, PDT has been applied to other hard-to-treat diseases such as rheumatoid arthritis, actinic keratosis, and bacterial infections. Researchers have used PDT for antibacterial activity owing to its low toxicity, the negligible chance of drug resistance with mild adverse reactions, and excellent antibacterial potential.

#### 2.2.1. Mechanism of PDT

The primary mechanism of PDT relies on the generation of ROS, including hydroxyl radicals (·OH), singlet oxygens (^1^O_2_), or superoxide radicals (·O^2−^) after photoresponsive materials are exposed to laser irradiation [29]. It is widely acknowledged that “toxic” cellular waste (i.e., ROS) can permanently damage macromolecules such as nucleic acids, lipids, and proteins after entering bacterial cells. The ROS generated by the PDT can directly or indirectly interfere with the physiological activities of the cells, ultimately leading to cell death [30]. In a different procedure, ROS can attach to bacterial cell walls and membranes, leading to cell death. As illustrated in Figure 1, photocatalysts, photosensitization, surface plasmon resonance (SPR), heterojunctions, and up-conversion luminescence routes can be utilized under light irradiation for PDT-mediated antibacterial activity.

The basic antibacterial mechanism of photocatalysts, such as ZnO [31] and TiO_2_ [32], depends on the nature of the ROS produced during the photoexcitation process. As illustrated in Figure 1, after photoirradiation, the excited electrons (e^−^) on the CB reduce O_2_ into ·O^2−^. Similarly, the photoinduced holes (h^+^) on the VB oxidize H_2_O to ·OH. These ROS can be further converted to other types of ROS for antibacterial activity [17]. Another effective approach for ROS generation is to form heterojunctions with two or more semiconductors. This can promote charge separations and transfers in single semiconductors instead of the rapid recombination of electron–hole pairs [33], eventually stimulating ROS production and antibacterial activity.

The ROS generation mechanism for PSs differs from that for photocatalysts [34]. There are two main molecular-level mechanisms in the photosensitization route [35] for controlling PDT-mediated antibacterial activity (Figure 1). One of the mechanisms normally occurs in bacterial cell membranes. In this mechanism, molecules from the PS temporarily migrate from the ground state to the singlet state (^1^PS) and subsequently reach the time-extended triplet state (^3^PS) by inter-system scurrying. In this scenario, the ^3^PS produces ROS, such as ·OH and ·O^2−^, through electron transfer after reacting with biomolecules in the surrounding environment. Subsequently, the ROS disrupt the bacterial cell membranes and increases their ion permeability for killing bacteria. In the second mechanism, the ^3^PS can directly react with oxygen molecules to undergo an energy transfer for the formation of ^1^O_2_ with a very short lifetime (and only able to react within a micrometer range of its generation site). This singlet oxygen can cause oxidative damage to intracellular bacterial compounds and eventually kill them. The PS molecules return to their ground states for the next cycle of reactions after the reactions are complete.

SPR also has the potential to enhance PDT-mediated antibacterial activity [36]. Photoinduced noble metals (Ag and Au) and metallic compounds (CuS) can produce SPR, which activates electrons that can be transferred to the CBs of the photocatalysts (Figure 1). As a second mechanism, the irradiation near the plasmon resonance frequency of noble metals can substantially enhance the local electric field, accelerating the separation of e^−^-h^+^ pairs. In this regard, PSs and noble metals can be applied together for better antibacterial activity [37].

Up-conversion nanoparticles (UCNPs) represent another approach to resolving the poor penetration ability of PDT. UCNPs can convert the excitation of long-wavelength light into short-wavelength light for PDT based on visible light-responsive photocatalysts [38].

#### 2.2.2. Advantages of PDT

Researchers are eager to seek alternatives to antibiotics owing to the ever-increasing number of MDR pathogens. In this regard, PDT has emerged as a viable option for treating MDR pathogens, owing to the following advantages: (1) broad-spectrum activity with ROS production, which disrupts many metabolic pathways of bacteria and their cellular structures rather than focusing on a single process or structure; (2) light-irradiation-induced antibacterial activity with photoresponsive ROS-generating materials; correspondingly, the chances of bacteria becoming resistant to the photoresponsive materials are very minimal; (3) preferential control of the binding to bacteria at the infected site of the body, light time, and location [39]; (4) low cytotoxicity to normal cells when treating bacterial infections in living organisms; (5) combinations with other therapies, such as radiotherapy, chemotherapy, and PTT; and (6) remote chances of any self-adaptability transferring to the next generation of bacterial cells in a very short time. Hence, the chances of bacteria becoming resistant are greatly reduced.

#### 2.2.3. Limitations of PDT

Despite the above-mentioned advantages of PDT, certain limitations must be addressed before it can be fully utilized to treat bacterial infections in living organisms. ROS-mediated PDT depends on photoresponsive materials and a light source for the irradiation of biological tissues. However, owing to the thickness of human tissue, the poor penetration ability or shallow depth of short-wavelength light or both has hindered the application of PDT to human tissues for antibacterial infections [40]. A longer-wavelength light source could provide greater tissue penetration. However, the ROS production for PDT is directly correlated with the energy of the light; therefore, light sources with longer wavelengths and low energies limit the production of ROS. Thus, UV light with a short wavelength and high energy can produce more ROS and is highly effective in killing microorganisms [41]. However, UV irradiation can also damage normal cells and tissues. Hence, the selection of an appropriate light source must be addressed to improve PDT applications.

Additionally, the development of PDT for clinical use is constrained by the limited release distance and lifespan of the ROS, as well as the low stability, toxicity, and ineffective bacterial targeting of certain photoresponsive materials [17]. Notably, PDT is less effective for G− bacteria than for G+ bacteria owing to the different membrane structures and limited penetration ability of ROS; this also requires further research. Therefore, the limitations of PDT need to be fully addressed before it can be further applied in practical applications.

## 3. Promising Aspects of Synergistic Action of PTT and PDT Combination

From the information on PTT and PDT discussed above, individual PTT and PDT for antibacterial activity have certain limitations hindering their potential growth for broad-scale and/or clinical application. To overcome this, researchers have attempted to improve the functionality of the individual strategies to resolve the limitations of PTT (for example, higher temperature requirements) and PDT (for example, limited tissue penetration) for excellent antibacterial activity. Notably, however, a synergistic combination of PTT and PDT has recently emerged with excellent potential to overcome the individual disadvantages of PTT and PDT [12,17,20]. The synergistic strategy is quite simple, as the combination enhances the antibacterial potential and retains the potential advantages of each process and compensates for the deficiencies of each strategy [42].

To understand the synergistic mechanisms of PTT and PTT, we must first understand the individual mechanisms of PTT and PDT, as discussed above. In PDT, photoresponsive materials are irradiated by light energy, generating ROS, and quickly killing bacteria. However, a single photodynamic agent requires large amounts of ROS to fulfill the requirements of PDT and kill bacteria. The main drawback, in this case, is that a large amount of ROS also causes problems for normal cells, such as inflammation and fibrosis [43]. To counter this problem, PTT can be employed together for better efficiency for both processes. The heat generated by the PTT can reduce the cell activity of the bacteria and make them more sensitive to ROS, correspondingly reducing the amount of ROS required. Similarly, the higher temperature requirement of PTT to kill bacteria if utilized alone can be reduced if used with PDT, as the ROS generated from the PDT process damages the bacteria and reduces the singular burden of the high-temperature generation by the PTT. Following a similar strategy and synergistic mechanisms, the higher dosage requirements for PTAs could be reduced.

Similarly, the weak penetration ability of ROS can be improved using PTT. As discussed above, the ROS production for PDT directly depends on the energy of the light source, where a higher-energy light source can produce more ROS but reduces the chances of tissue penetration owing to the shorter wavelength [40]. UV light has mostly been used to produce more ROS and kill microorganisms owing to its short wavelength and high energy. However, it also runs the risk of damaging normal healthy tissues because of the negative effect of UV light on mammalian cells [44]. In this scenario, PTT and PDT can be conducted together to achieve synergistic activity. The synergistic mechanism of this process can be realized by the fact that the PTT with the longer-wavelength light source and low energy can penetrate easily to the infection site and induce heat to improve the permeability of the pathogenic cell membrane, which in turn enables easier cellular permeation of the ROS into the membrane and compensates for the lower ROS permeability for PDT [45]. Simultaneously, the ROS can also reduce the heat resistance of the bacteria, thereby reducing the higher temperature requirements for PTT efficacy.

Various researchers have utilized several nanomaterials to achieve synergistic PTT- and PDT-based antibacterial applications (Table 1). Carbon-based materials have shown excellent potential for antibacterial activity as photoresponsive materials [46]. For example, in one study, carbon-based materials were utilized for dual-modal PTT and PDT owing to their potential for hyperthermia and ROS generation through light activation [47]. Zhang et al. [48] utilized cationic carbon dots (CCDs) on black phosphorus (BP) nanosheets, which could bind to the anionic sites of bacteria via electrostatic attraction. BP@CCD produced BP-derived photothermal effects and CCD-originating photodynamic effects in response to mixed laser radiation (660 and 808 nm) for synergistic antibacterial activity. Similarly, graphene quantum dots (GQDs) have been synergistically utilized with germicidal chitosan oligosaccharide (COS) for the simultaneous production of ROS and hyperthermia after 450 nm visible light irradiation, providing highly efficient removal of bacterial infections both in vitro and in vivo [49].

PSs have also been used with PTAs for synergistic PDT with PTT. For example, graphene nanoribbons (GNRs) as PTA have been applied with polycationic porphyrin (Pp4N) as the PS for hyperthermia generation along with the electrostatic interactions between the cationic Pp4N and negatively charged bacterial cells for wide-spectrum drug-resistant antibacterial activity [50]. In a similar experiment, novel polyfluoroalkyl-substituted silicon phthalocyanine and single-walled carbon nanotubes (SWNTs) were utilized together for heat generation and ROS production after light irradiation and showed synergistic antibacterial activity [51]. Similarly, indocyanine green (ICG) loaded 3-aminopropylsilane coated superparamagnetic iron oxide nanoparticles (APTMS@SPIONs) were evaluated on planktonic cells and biofilms of G- (*Esherichia coli*, *Klebsiella pneumoniae*, *Pseudomonas aeruginosa*) and G+ (*Staphylococcus epidermis*) bacteria for synergistic PTT- and PDT-based antibacterial activity [52]. Cai et al. [53] also encapsulated ICG and manganese pentacarbonyl bromide (MnBr(CO)_5_) into a 2,2′-bipyridine-4-carboxylic (BPY)-modified peptide dendrimer-based nanogel (G3KBPY) for ICG&CO@G3KBPY nanocomposite which was experimented to treat biofilm infection by synergistic PTT and PDT.

Nanomaterial-based dual-modal PTT and PDT approaches have also been explored for synergistic antibacterial activity. In various studies, gold nanoparticles and polydopamine (PDA)-loaded hydroxyapatite [54], a poly(5-(2-ethylacrylate)-4-methylthiazole-g-butyl)/copper sulfide (CuS) cluster [55], Ce6 combined with ultrasmall protein-modified CuS nanoparticles [56], and CuS-BP nanocomposites [57] have shown PTT and PDT effects under NIR laser irradiation for effective antibacterial activity. Polyethylene glycol-functionalized poly(N-phenylglycine) nanoparticles (PNPG-PEG NPs) also showed an excellent synergistic PTT and PDT potential for antibacterial activity against *E. coli* and *Staphylococcus aureus* [58]. Different small organic molecules were also explored for synergistic PTT- and PDT-based anti-cancer activity [59,60,61], but not much for antibacterial activity.

The approach to PTT and PDT combination therapy should be synergistic and not only a one-plus-one combination method. Hence, proper experimental procedures and functionalization must be performed to achieve the final synergistic goal of “1 + 1 > 2”.

**Table 1:** Photothermal therapy (PTT)- and photodynamic therapy (PDT)-based synergistic antibacterial applications.

Material	Light Source	Microorganisms	Ref.
BP@CCD	660 and 808 nm	*E. coli, S. aureus*	[48]
GQDs-COS	450 nm	*E. coli, S. aureus*	[49]
Pp4N/GNR	660 and 808 nm	*A. baumannii*, MRSA	[50]
SiPc-F-SWNTs	680 nm	*E. coli*	[51]
ICG loaded APTMS@SPIONs	808 nm	*E. coli, K. pneumoniae, P. aeruginosa, S. epidermidis*	[52]
PDA@Au-HAp	808 nm	*E. coli, S. aureus*	[54]
PATA-C_4_@CuS	980 nm	Levofloxacin-resistant *S. aureus, E. coli, P. aeruginosa, B. amyloliquefaciens*	[55]
Ce6-BSA-CuS NPs	660 and 1064 nm	*E. coli, S. aureus*	[56]
PNPG-PEG NPs	810 nm	*E. coli, S. aureus*	[58]
CuS nanostructures	808 nm, Simulated solar light	*E. coli*	[62]
CuS nanoparticle complex hydrogels	808 nm	*E. coli, S. aureus*	[63]
CuS nanodots	808 nm	β-lactamase *E. coli*, MRSA	[64]
PDA-Cur	405 and 808 nm	*E. coli, S. aureus*	[65]
Fe_3_O_4_–Au–PDA	808 nm	*E. coli*	[66]
BC/MoS_2_-CS	≥420 nm, xenon lamp	*E. coli, S. aureus*	[67]
MoS_2_@Au nanorods	808 nm, xenon lamp	*E. coli*	[68]
UCNPs@TiO_2_@GO-PVDF	980 nm	*E. coli, S. aureus*	[69]
GO-tobramycin@CuS	980 nm	Antibiotic-resistant *P. aeruginosa, S. aureus*	[70]
GO/nGO	630 nm	*E. coli, S. aureus*	[71]
ICG-GO	808 nm	MRSA	[72]
VCL/PEGDA-MNPs-MWCNTs-ZnMintPc	630 nm	*E. coli, S. aureus, C. albicans*	[73]
nGO-BSA-AIEgen	795 nm	AMO-resistant *E. coli, S. aureus*	[45]
BP@Te doped CDs	808 nm	*E. coli, S. aureus*	[74]
N doped CDs@Cur	405 and 808 nm	*E. coli, S. aureus*	[75]
Cu-CDs-C35	808 nm	*E. coli, S. aureus*	[76]
GQD–AgNP	450 nm	*E. coli, S. aureus*	[77]
DMCPNs	808 nm, White light	Ampicillin-resistant *E. coli*	[78]
PEDOT:ICG@PEG-GTA	808 and 1064 nm	*E. coli, S. aureus*	[79]
Ti-RP/PCP/RSNO	808 nm	MRSA	[80]
Ti-PDA/ICG/RGD	808 nm	*S. aureus*	[81]
AuNRs@Cur	405 and 808 nm	*E. coli, S. aureus*	[82]
Au@Bi_2_S_3_	808 nm	*E. coli, S. aureus*	[83]

Abbreviations: *A. baumannii*, *Acinetobacter baumannii*; *B. amyloliquefaciens*, *Bacillus amyloliquefaciens*; BP, Black Phosphorus; *C. albicans*, *Candida albicans*; CCD, cationic carbon dots; GQDs, graphene quantum dots; COS, chitosan oligosaccharide; Pp4N, polycationic porphyrin; GNR, graphene nanoribbon; MRSA, Methicillin-resistant *S. aureus*; SiPc-F, polyfluoroalkyl substituted silicon phthalocyanine; SWNTs, single wall carbon nanotubes; ICG, indocyanine green; APTMS, 3-aminopropylsilane; PDA, polydopamine; Hap, hydroxyapatite; PATA,C_4_- poly(5-(2-ethyl acrylate)-4-methylthiazole-*g*-butyl); PNPG, poly(*N*-phenylglycine); Cur, Curcumin; BC, bacterial cellulose; CS, chitosan; GO, graphene oxide; UCNP, up-conversion nanoparticle; PVDF, poly(vinylidene) fluoride; VCL/PEGDA, poly (N-vinylcaprolactam-co-poly(ethylene glycol diacrylate)) poly (VCL-co-PEGDA) polymer; MNPs, iron magnetic nanoparticles; MWCNTs, multiwall carbon nanotubes; ZnMintPc, menthol-Zinc phthalocyanine; AIE, aggregation-induced emission fluorogen; AMO, amoxicillin; CDs, carbon quantum dots; C35, cocoamidopropyl betaine; DMCPNs, dual-mode conjugated polymer nanoparticles; PEDOT, poly(3,4-ethylenedioxythiophene) nanoparticle; GTA, glutaraldehyde; Ti-RP, red phosphorus modified Ti implants; PCP, PVA/CS/PDA hydrogel; RSNO; S-nitrosuccinic acid.

### Synergistic PTT and PDT Agents

To overcome the individual limitations of PTT and PDT, researchers have utilized dual-mode synergistic PTT- and PDT-based antibacterial therapies. In this respect, research works based on synergistic PTT- and PDT-based antibacterial activity have mostly combined PTT and PDT agents [50,55,56]. The purpose of this conceptual approach to research was to exploit the advantageous properties and overcome the limitations of each process for synergistic antibacterial activity. In one study, owing to the absorption mismatch between the photoresponsive agents for individual PTT and PDT, two different wavelengths of light sources were applied in a nanocomposite to excite PSs and PTAs, respectively [50,56]. Despite obtaining impressive outcomes after exploiting this concept, certain limitations must be resolved. (1) Utilizing both agents together and irradiation with different wavelengths of light is economically costly. (2) The synthesis process for the combination of PTT and PDT is more complex than the individual processes. (3) Proper functionalization is needed for nanocomposite formation with PTT and PDT agents because the various physiochemical characteristics (for example, surface potential, solubility, stability, toxicity, and biodegradability) can interfere with nanocomposite formation and eventually hinder the outcome.

Hence, combining a single nanomaterial or nanocomposite with NIR light irradiation can represent a potential solution to the aforementioned problems, where the heat generation for PTT and ROS production for PDT can be achieved using a single photoresponsive nanomaterial or nanocomposite with a single NIR light source. Notably, the penetration ability of NIR light is far greater than that of UV or visible light [84] and can be utilized by photoresponsive materials to generate ROS and increase temperatures for antibacterial or anti-cancer activity [63,64]. Moreover, NIR light has clinical potential considering its anti-cancer or antibacterial activity [21,28]. Hence, it is easy to understand the extensive use of NIR light not only for antibacterial activity but also for other biomedical applications. Therefore, the aim of researchers in recent years has been to synthesize nanomaterials with the ability of NIR light-mediated photothermal conversion as well as the capacity for singlet oxygen generation [63,64]. Although research in this direction is scarce, there are some materials, such as CuS, PDA, and molybdenum disulfide (MoS_2_), which show potential for use in further research regarding NIR light-mediated synergistic PTT- and PDT-based antibacterial activity.

CuS, a semiconducting metal chalcogenide with a narrow bandgap, is widely known as a photothermal nanomaterial with anti-cancer and antibacterial activities owing to its excellent NIR light absorption properties [85]. As discussed above, CuS can absorb NIR light using the LSPR originating from the collective oscillation of holes instead of the electron-mediated LSPR for noble metals [85]. Therefore, unlike noble metals, the LSPR position of CuS (and, accordingly, its photothermal properties) can be controlled by modulating the number of carriers. Moreover, CuS shows the ability to generate ROS after irradiation with NIR light. Thus, it can be functionalized for use as a PTT- and PDT-based synergistic antibacterial agent without the need for individual materials. For example, Mutalik et al. [62] demonstrated the morphology-dependent photodynamic and photothermal antibacterial activity of NIR light-irradiated CuS against *E. coli*. Similarly, Zhou et al. [63] demonstrated an injectable self-healing hydrogel system consisting of a CuS-based complex with the potential to completely eradicate *E. coli* and *S. aureus* via synergistic PTT and PDT under NIR laser irradiation. Qiao et al. [64] demonstrated a similar NIR laser-irradiated PTT- and PDT-based antibacterial potential of CuS nanodots against methicillin-resistant *S. aureus* (MRSA) and extended-spectrum β-lactamase *E. coli.*

PDA is another material with excellent PTT- and PDT-based synergistic potential for NIR light-mediated antibacterial activity [86]. Several advantageous properties of PDA, such as its simple preparation process, easy functionalization, excellent photothermal and photoacoustic properties, biocompatibility, and biodegradability, have led to several PDA-based biomedical research studies. Regarding antibacterial activity, PDA is regarded as an excellent PTA with a good ROS generation ability after laser irradiation. Hence, a single nanomaterial or nanocomposite can be used for synergistic PTT and PDT after NIR light irradiation using PDA. Su et al. [65] demonstrated the excellent PTT- and PDT-based synergistic antibacterial activity of PDA-based nanocomposites. PDA–curcumin (PDA–Cur) nanocomposites) were prepared via self-polymerization. The PDA–Cur nanocomposites showed good antibacterial activity against *S. aureus* and *E. coli* with good biocompatibility. In another experiment, magnetic Fe_3_O_4_–Au–PDA hybrid microcapsules were developed for NIR laser-irradiated synergistic PTT- and PDT-based antibacterial activity against *E. coli* [66].

MoS_2_ is another nanomaterial that has shown significant potential in this category. Similar to PDA, MoS_2_ is an excellent PTA material with remarkable photothermal antibacterial properties and has the potential to generate ROS after laser irradiation. Hence, synergistic PTT- and PDT-based antibacterial activity is possible with MoS_2_-based nanocomposites. Shen et al. [67] developed in situ-grown bacterial cellulose/MoS_2_–chitosan nanocomposite materials for PTT- and PDT-based antibacterial activities. The nanocomposite showed excellent antibacterial efficacy against *E. coli* (99.998%) and *S. aureus* (99.988%) after visible-light irradiation. Yougbaré et al. [68] also demonstrated antibacterial activity against *E. coli* like that of MoS_2_@Au nanorods with NIR laser irradiation.

## 4. Synergistic Antibacterial Applications of PTT and PDT

Synergistic combination therapies of PTT and PDT have been designed based on multi-target bactericidal activity, where the deficiencies of a single treatment therapy are overcome via the synergistic combination. Researchers have tested various materials for synergistic antibacterial activity. In this review, we summarized (1) carbon-, (2) polymer-, and (3) metal-based materials for synergistic antibacterial PTT and PDT combinations.

### 4.1. Carbon-Based Combination of PTT and PDT

Carbon-based materials have been considered ideal for various biomedical applications owing to their negligible cytotoxicity and environmentally benign nature [87,88]. Additionally, their tunable parameters (such as size, shape, and layer number) can generate excellent physiochemical properties and have proven excellent assets for antibacterial activity [49,50,70,71,72,87]. Typically, graphene, carbon dots, and carbon nanotubes (CNTs) have been explored for synergistic PTT and PDT therapies owing to their NIR light absorption properties and photothermal conversion abilities.

The immense success of graphene in biomedical applications is well-known, with numerous studies on graphene as a core material [70,71,72,87,88]. The unique optical properties of graphene and its derivatives \[graphene oxide (GO) and reduced GO (RGO)], that is, their ability to absorb light energy and release it as heat energy, have encouraged researchers to utilize it for PTT applications [70,71,72]. Furthermore, graphene can produce ROS after irradiation with NIR light. It is also believed that 2D graphene can utilize its sharp edges to destroy the cellular membranes of bacteria, ultimately killing them [89]. The broad-spectrum antibacterial activities of GO and RGO have also been exploited by researchers [70,71,72,87].

**Figure 2:**
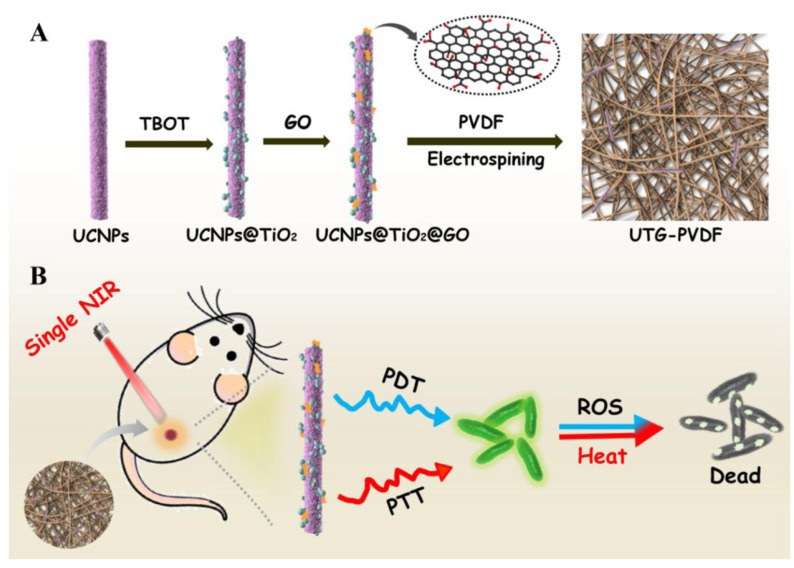
(**A**) Schematic diagram of up-conversion nanoparticles@TiO_2_@graphene oxide (UTG)-poly(vinylidene) fluoride (PVDF) nanocomposite membrane synthesis, (**B**) Near-infrared (NIR) light-mediated PTT- and PDT-based synergistic antibacterial activities of UTG-PVDF membrane. Reproduced with permission from Ref. [69]. Copyright 2019, American Chemical Society.

Another advantage of graphene is that it can be functionalized into a single nanocomposite for synergistic photothermal and photodynamic antibacterial activity using a single NIR light source. For example, Sun et al. [69] reported the utilization of up-conversion nanoparticles (UCNPs) consisting of NaYF_4_:Yb:Tm nanorods with a hierarchical core-shell structure (UCNPs@TiO_2_) (Figure 2). Subsequently, photothermal GO was incorporated into the UCNPs@TiO_2_ for a UCNPs@TiO_2_@GO (UTG) nanocomposite. Finally, the mixture was electrospun in a poly(vinylidene) fluoride (PVDF) membrane to prepare a UTG–PVDF nanocomposite. A single NIR light (980 nm) source was used to irradiate the UTG–PVDF nanocomposites for 5 min. Simultaneously, ROS were generated, and the temperature increased, providing synergistic antibacterial effects against both G- and G+ bacteria. This antibacterial activity is difficult to achieve using individual photodynamic or photothermal nanocomposite membranes. Furthermore, the graphene-based nanocomposite membrane accelerated wound healing, further enhancing its potential for infectious wound healing studies.

The NIR light absorption of graphene has been frequently used for heat energy generation and ROS production [49,50,70,71,72]. However, the fast recombination rate of the electrons and holes in GO hinders its application in synergistic PTT and PDT. Dai et al. [70] found a solution to this problem by introducing CuS and the aminoglycoside antibiotic tobramycin (Tob) to form a GO–Tob@CuS hybrid nanoplatform. CuS was added to solve the problem of fast recombination of the electrons and holes. Additionally, the broad-spectrum antibiotic Tob was selected to modify the surface charge potential of the GO because the surface charge of GO is usually negative and is not ideal for interactions with electronegative bacterial surface charges. The GO–Tob@CuS nanocomposite showed excellent PTT and PDT effects after NIR laser (980 nm, 1.5 W·cm^−2^) irradiation for 5 min, with a temperature increment of up to 65 °C at a concentration of 100 μg·mL^−1^. ROS were also produced after the NIR irradiation. Moreover, the GO–Tob@CuS nanocomposite showed irreversible damage to antibiotic-resistant *P. aeruginosa* and *S. aureus* species after NIR laser irradiation by utilizing the nano-knife effect along with the PTT and PDT properties of the nanocomposite.

In another experiment, Romero et al. [71] demonstrated the broad-spectrum and selective antibacterial activity of GO and nanographene oxide (nGO) against *E. coli* and *S. aureus* species by utilizing the synergistic PTT and PDT effects. The GO and nGO showed the ability to form ROS with an increase in temperature from 55 to 60 °C when irradiated with an LED red light at 630 nm (65 mW·cm^−2^), effectively killing the targeted bacterial cells using the synergistic PTT and PDT effects. In another direct synergistic procedure, the PS indocyanine green (ICG), usually used for PDT, was loaded onto photothermal GO nanosheets for NIR-irradiated anti-MRSA activity [72]. The NIR (808 nm, 1 W·cm^−2^)-irradiated ICG–GO showed a photothermal temperature increment of 43.1 °C and the highest ROS generation (increments of 316.51% and 157.11% compared with ICG and GO alone, respectively), representing a potent material for inhibiting the growth of MRSA. In a similar study, the PS menthol-zinc phthalocyanine was encapsulated in magnetic nanocomposites with GO and multiwall CNTs to prepare a biocompatible hydrogel [73]. Subsequently, its broad-spectrum antibacterial activity was evaluated based on its synergistic PTT and PDT effects. The magnetic nanocomposite was irradiated with a red light of 630 nm/65.5 mW·cm^−2^. The nanocomposite was able to eliminate three types of microbial colonies (*C. albicans*, *E. coli*, and *S. aureus*) using the synergistic PTT and PDT effect and could be considered a broad-spectrum photoresponsive antibacterial agent. GNRs [50] and SWNTs [51] have also been utilized for synergistic antibacterial activities. Zhang et al. [45] entrapped hydrophobic aggregation-induced emission fluorogens (AIEgens) in amphiphilic nGO and bovine serum albumin (BSA) nanocomposites. The biocompatibility of the nanoparticles was enhanced by conjugation with the BSA. Moreover, the nanocomposite formed a dual-mode (PTT and PDT) antibacterial application using the heat produced by the nGO- and AIEgen-produced ROS for collaborative antibacterial results under light irradiation.

Despite the immense success of graphene in biomedical applications (including those based on antibacterial activity), many hurdles remain before it can be fully applied in clinical applications. For example, the complex interactions of graphene with biological membranes can lead to endotoxin contamination during synthesis, mitochondria-mediated apoptosis, and septic shock [90]. In this regard, researchers have developed carbon quantum dots (CDs), a new carbon-based nanomaterial for biomedical applications [74,75,76]. CDs are well-known for their biocompatibility, excellent water solubility, and stability. However, the NIR absorption properties of CDs are not satisfactory for phototherapy. Therefore, more functionalized CDs for CD-based nanocomposites are required for the synergistic PTT and PDT effect to achieve antibacterial activity.

Liu et al. [74] constructed a 2D antibacterial nanoplatform by combining liquid-phase exfoliated BP and hydrothermally prepared tellurium-doped CDs, thereby combining the favorable biomedical properties of CDs with the photothermal properties of BP. A single 808 nm NIR laser irradiation process was used to induce the photothermal conversion of BP with ROS generation from the CDs. The BP@CDs showed PTT- and PDT-mediated excellent synergistic antibacterial activity against *E. coli* and *S. aureus* (as high as 98.4 and 92.7%, respectively), coupled with a faster wound closure ratio than other infected wounds. The BP@CD nanoplatform also showed good hemocompatibility, cytocompatibility, and biocompatibility; thus, it could be utilized clinically. Zhang et al. [48] performed a similar experiment using BP and CCDs.

**Figure 3:**
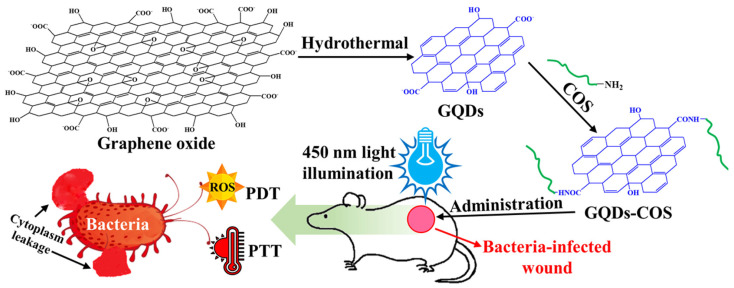
Schematic illustration of graphene quantum dots (GQDs)-chitosan oligosaccharide (COS) synthesis and its PTT- and PDT-based synergistic antibacterial wound healing. Reproduced with permission from Ref. [49]. Copyright 2020 American Chemical Society.

In another study, nitrogen-doped CDs were compounded with Cur to prepare a synergistic nanocomposite showing dual-mode PTT and PDT antibacterial activity [75]. In this synergistic nanocomposite, the biocompatibility and rich amphiphilic functional group-mediated solubility of the CDs perfectly complemented the Cur, a natural PS with powerful antibacterial properties but with insolubility in water. Blue (405 nm) and near-infrared (808 nm) light sources were utilized for generating ROS and hyperthermia, respectively. The CD-based Cur nanocomposite showed synergistic PTT- and PDT-based antibacterial activity against *E. coli* and *S. aureus* under the dual light source (405 + 808 nm) irradiation, along with low toxicity and good hemocompatibility. A similar approach was reported by Mei et al. [49], in which COS-functionalized GQDs were utilized to kill *E. coli* and *S. aureus* after short-term exposure to 450 nm visible light (Figure 3). The nanocomposite also showed good hemocompatibility and low cytotoxicity for infected wound healing.

Metal-based nanoparticles also have antibacterial and excellent NIR absorption properties, with numerous reports supporting these claims [91,92,93]. However, their toxicity hinders their further growth. A combination of metal nanoparticles with CDs reduces the risk of toxicity owing to the CDs and increases the chances of obtaining photothermal properties owing to metal-based nanoparticles. For example, Chu et al. [76] proposed a combination of copper ions with CDs and quaternary amino compounds for synergistic photothermal and photodynamic-based antibacterial activity against *E. coli* and *S. aureus*. with 808 nm NIR light irradiation, leading to a temperature increment of up to 57.3 °C and efficient ROS production. Thus, a synergistic PTT and PDT approach was established. In another similar experiment [77], Ag-conjugated GQDs were utilized as blue light-enhanced nanotherapeutics for antimicrobial therapy. The nanocomposite showed improved production of ROS combined with a significant increase in temperature for synergistic PTT- and PDT-mediated antibacterial activity.

### 4.2. Polymer-Based PTT and PDT

Conjugated polymers (CPs) have shown excellent potential for biomedical applications owing to their unique conjugated backbone structures and exceptional photophysical properties [78,79]. One of the major advantages of CPs is that they can generate ROS and heat based on light energy irradiation after synthesis by regulating the backbone structures. Additionally, CPs are biocompatible and photostable, and the cost of their synthesis is low. Unsurprisingly, CPs have emerged as potential candidates for PSs (PDT) and PTAs (PTT). Moreover, water-insoluble CPs can be developed into nanoparticles with good dispersibility and biocompatibility for use in aqueous media without complicated water-soluble modifications [94]. These water-insoluble CPs can also be utilized for the photothermal releases of drugs. Researchers are constantly trying to exploit the beneficial properties of PTT and PDT while limiting the deficiencies of each process. CPs may represent a valid force for synergistic PTT- and PDT-mediated antibacterial activity.

Cui et al. [95] developed a water-insoluble conjugated polymer (PDPP)-mediated antibacterial hydrogel with synergistic PTT and PDT antibacterial capabilities. A nanoprecipitation method was used to convert the PDPP into water-insoluble nanoparticles. Subsequently, the cell-penetrating peptide “TAT” was grafted onto the PDPP surface. Finally, a polyisocyanide (PIC) hydrogel was added. The PIC hybrid intelligently regulated its antibacterial ability when sequentially irradiated with white light and NIR light and showed stronger antibacterial ability than PDT or PTT alone.

**Figure 4:**
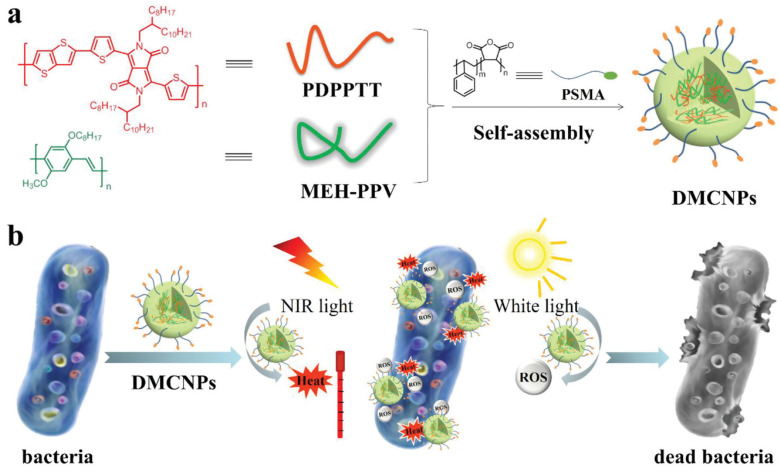
(**a**) Schematic illustration for the synthesis of utilized dual-mode antibacterial conjugated polymer nanoparticles (DMCPNs), (**b**) PTT- and PDT-based synergistic antibacterial activity of DMCPNs. Reproduced with permission from Ref. [78]. Copyright 2019 Wiley.

In another study, Zhang et al. [78] utilized dual-mode antibacterial conjugated polymer nanoparticles (DMCPNs) for PTT and PDT for the efficient killing of ampicillin-resistant *E. coli* (Figure 4). A co-precipitation method was employed for the self-assembly of DMCPNs using poly(diketopyrrolopyrrole-thienothiophene) as the PTA and poly [2-methoxy-5-((2-ethylhexyl)oxy)-p-phenylenevinylene] as a PS. Poly(styrene-co-maleic anhydride) has also been used for nanoparticle dispersion in water via hydrophobic interactions. The DMCPNs produced a 93% inhibition rate of ampicillin-resistant *E. coli*. after dual irradiation with near-infrared light (550 mW·cm^−2^, 5 min) and white light (65 mW·cm^−2^, 5 min). This antibacterial activity was attributed to the ability of DMCPNs to generate photothermal effects and ROS after exposure to oxygen in the surroundings after light irradiation.

Despite the excellent potential of CPs for PTT- and PDT-mediated antibacterial activity, more CP nanoparticles absorbed NIR light in the first biological window (NIR-I, 700 and 950 nm) than in the second NIR window (NIR-II, 1000 and 1250 nm) [96]. The NIR-II window has advantages regarding the spatial scattering effect and penetration depth. Following this principle, Li et al. [79] synthesized a poly(3,4-ethylenedioxythiophene) nanoparticle (PEDOT)-based nanocomposite for dual-mode (PTT + PDT) antibacterial activity owing to the broad absorbance of PEDOT in the NIR region (700 and 1250 nm). In this work, ICG dye was conjugated with PEDOT using an environmentally friendly hydrothermal method utilizing the electrostatic attraction between them. The PEDOT nanoparticles were initially modified with glutaraldehyde (GTA) for bacterial targeting. The functionalized and stable PEDOT:ICG@PEG-GTA nanocomposite exhibited excellent synergistic antibacterial potential against pathogenic bacteria in the presence of NIR irradiation (808 nm for PDT and 1064 nm for PTT) in both the NIR-I and -II windows and was considerably more effective than PTT or PDT alone.

An MRSA infection through biofilm was also managed using a hydrophilic and viscous hydrogel consisting of PVA modified with chitosan, PDA, and an NO release donor formed on a red phosphorus nanofilm deposited on a titanium (Ti) implant. The MRSA biofilm was synergistically destroyed by ROS and hyperthermia under NIR irradiation [80]. Yuan et al. [81] exploited NIR light-triggered PTT and PDT to eradicate biofilms on Ti implants. Mesoporous PDA (MPDA) nanoparticles were immobilized onto an amino-modified Ti surface. An ICG PS was then integrated into the MPDA by π–π stacking. Finally, to eliminate the NIR-triggered *S. aureus* biofilm in vivo through the synergistic PDT and PTT effects, the system was functionalized with an RGD peptide for the final sample (Ti-M/I/RGD).

### 4.3. Metal-Based Combination of PTT and PDT

Metal-based nanoparticles are well-known for their inherent antibacterial properties. Au, Ag, Pd, ZnO, CuO, and CuS have been reported to exhibit antibacterial activities owing to their favorable physicochemical properties [1,97]. Moreover, some of them are considered photoresponsive materials due to their ability to absorb light sources and convert them into heat energy or to produce ROS after light irradiation [85,98]. However, their poor photostability, low targeting efficiency, and cytotoxicity in mammalian cells have hindered their broad application for clinical antibacterial activity. Hence, researchers are constantly trying to exploit the advantageous properties of metal-based nanoparticles for PDT- and PTT-based antibacterial activity in combination with efforts to limit their deficiencies.

**Figure 5:**
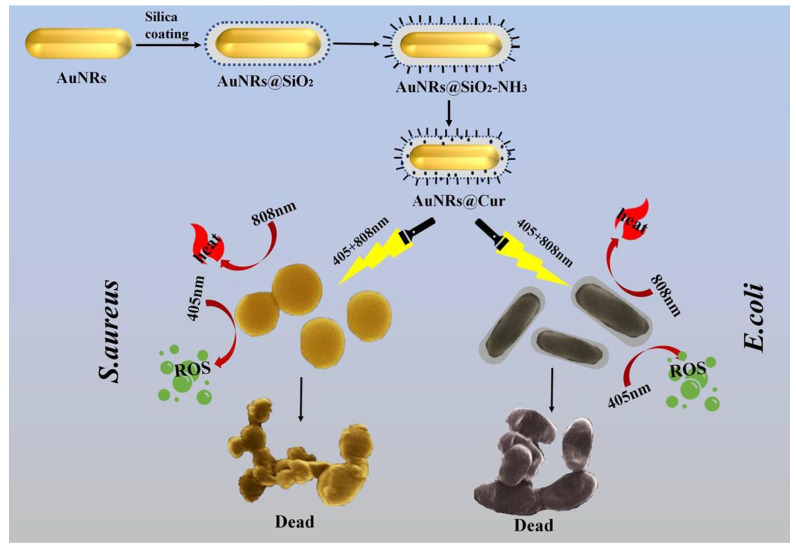
Schematic illustration of preparation and synergistic antibacterial activity of gold nanorods (Au NRs)@curcumin (Cur). Reproduced with permission from Ref. [82]. Copyright 2022 Elsevier.

Zhang et al. [82] synthesized core-shell gold nanorod (Au NR)@mesoporous-silica/Cur nanocomplexes for dual-mode PTT and PDT antibacterial activity (Figure 5). Each material in the nanocomposite had a specific role, with the overall goal being synergistic PTT- and PDT-mediated antibacterial activity. Au NRs have been intentionally exploited in gold nanocrystals owing to having the highest photothermal ability. In one study, cationic surfactant cetyltrimethylammonium bromide (CTAB) was used to prepare Au NRs and was essential to improving their stability. However, CTAB also has low compatibility and high toxicity in molecular biofilms. Hence, biocompatible mesoporous silica (SiO_2_) was employed to coat the Au NRs to remove the CTAB toxicity. Subsequently, Cur with antibacterial photodynamic potential was constructed onto the mesoporous silica modified Au NRs. The nanocomposite showed excellent dual-mode (PTT and PDT) antibacterial potential after double-wavelength (405 + 808 nm) laser irradiation. The biocompatibility of the nanocomposite was also appropriate for its potential in vivo application.

Similarly, another study successfully exploited sea urchin-like Au@Bi_2_S_3_ core-shell structures for the synergetic photothermal and photodynamic inactivation of *E. coli* and *S. aureus* after NIR light irradiation [83]. Fang et al. [66] synthesized a magnetic Fe_3_O_4_–Au–PDA hybrid microcapsule with both photothermal and photodynamic antibacterial potential. Later, tetracarboxyzinc phthalocyanine (ZnPc) was loaded into Fe_3_O_4_–Au–PDA hybrid microcapsules. The Au–PDA hybrid shell exhibited a photothermal nature and the photodynamic potential was served by the ZnPc PS. The final Fe_3_O_4_–Au–PDA/ZnPc hybrid microcapsule showed excellent synergistic (PTT and PDT) antibacterial activity against *E. coli* after irradiation with an 808 nm NIR laser.

Metal sulfides have recently emerged as exciting photothermal materials owing to their stability and NIR light-absorption properties [85]. Moreover, Cu-based nanomaterials have gained significant attention for their NIR-mediated antibacterial activity owing to their essential trace element status in the human body. Under these circumstances, CuS has emerged as an excellent NIR light-absorbing nanomaterial with PTT- and PDT-mediated antibacterial activity. For example, Dai et al. [55] prepared poly (5-(2-ethyl acrylate)-4-methylthiazole-g-butyl)/CuS nanoclusters to efficiently capture and eliminate levofloxacin-resistant G- and G+ bacteria using NIR light-mediated synergistic PTT and PDT. A thiazole derivative was applied to this nanocomposite as a membrane-targeting cationic ligand for the bacteria. Under NIR laser irradiation, these conjugated nanoclusters (980 nm, 1.5 W·cm^−2^, 5 min) significantly inhibited *B. amyloliquefaciens*, *E. coli*, levofloxacin-resistant *S. aureus*, and *P. aeruginosa* at 5.5 μg·mL^−1^. The generation of the heat and ROS after NIR light irradiation was attributed to the antibacterial activity, suggesting a synergistic PTT- and PDT-mediated antibacterial activity.

Wang et al. [99] reported that implant-related bacterial infections in orthopedic clinics could be overcome using dual-mode PTT and PDT. In one study, PEG-modified Cu_9_S_8_ nanoparticles with good biocompatibility were synthesized using a facile one-step wet chemical method. The PEG-modified Cu_9_S_8_ nanoparticles showed synergistic PTT- and PDT-mediated antibacterial activity with excellent antibiofilm activity against clinically collected pathogenic *S. aureus* growing on Ti surfaces under NIR irradiation (808 nm, 1 W·cm^−2^).

## 5. Conclusions and Future Perspectives

Notable developments have been made in killing bacteria and inhibiting their ever-increasing antibiotic resistance. However, it can also be unanimously agreed that a more precise and functionalized strategy is required to tackle MDR bacteria. Following this principle, synergistic PTT and PDT have the potential to be combined with antibiotics to offer a unique opportunity to effectively tackle infections caused by MDR bacteria. Additionally, the chances of bacteria becoming resistant to combination therapy are very remote owing to their non-invasive nature. A multimodal PDT and PTT combination strategy is also possible while using a single nanomaterial instead of using two materials for each individual property.

It is worthwhile to mention that thermal damage to normal tissues owing to the high temperature need of PTT may be managed by employing it with PDT therapy as it lowers the dependency on single-mode PTT use. Furthermore, the targeting ability of photoresponsive materials can be used to solve this problem. This targeting ability, however, necessitates additional research into the precise positioning of synergistic photoresponsive materials that can easily target the intended bacteria or infection site. In this respect, surface modification can be useful, as it is well-known that the surface of photoresponsive materials plays an important role in their antibacterial activity. Because most bacteria have a negative surface potential due to a peptidoglycan coating, electrostatic interactions may play a key role in this scenario. As a result, non-toxic positive charge molecules such as chitosan derivatives have been exploited for specifically targeting bacteria. However, further research is still required with respect to surface functionalization. Moreover, a more tailored synergistic antibacterial activity is required for the precision and accuracy of the antibacterial activity.

Additional PTT and PDT combinations may be relevant in future application-oriented research, such as drug administration and immunotherapy. Similarly, the limited penetration capacity of typical ROS light sources is easily overcome by high-penetration light sources of PTT when they are utilized synergistically. Furthermore, the ability of photoresponsive materials to penetrate the extracellular matrix of biofilm requires more research because bacteria can easily protect themselves within the extracellular matrix of biofilm due to limited options, as high concentrations of antibacterial agents induce cytotoxicity in normal cells. Some structural alteration, such as needle-like nanomaterials as synergistic PTT and PDT agents, is required in this respect. Instead of using nanocomposites, where different PTT and PDT agents are combined for synergistic antibacterial activity, more emphasis should be placed on finding single antibacterial agents, such as CuS, where the PTT- and PDT- based synergistic antibacterial activity can be experienced through a single nanomaterial.

Despite its remarkable potential, issues remain to be resolved. Certain clinical trials regarding the synergistic potential of PTT and PDT need to be established for practical applications. The photosensitive materials utilized for both PTT and PDT have not yet fully proven their non-toxicity. Greater research efforts are still required for photoirradiated synergistic PTT- and PDT-based antibacterial activity and can be further explored in clinical trials. In summary, we sincerely hope that the emergence of synergistic PTT and PDT will fulfill its potential and emerge as a serious force for clinically managing infections caused by MDR bacteria.

## Data Availability

Not applicable.
